# Epidemiology of Bacteremia in Young Hospitalized Infants in Vientiane, Laos, 2000–2011

**DOI:** 10.1093/tropej/fmt064

**Published:** 2013-07-31

**Authors:** Margot Anderson, Khonesavanh Luangxay, Kongkham Sisouk, Latdavan Vorlasan, Bandith Soumphonphakdy, Vanmaly Sengmouang, Vilada Chansamouth, Koukeo Phommasone, Russell Van Dyke, Euming Chong, David A.B. Dance, Rattanaphone Phetsouvanh, Paul N. Newton

**Affiliations:** ^1^ Lao-Oxford-Mahosot Hospital-Wellcome Trust Research Unit (LOMWRU), Microbiology Laboratory, Mahosot Hospital, Vientiane, Lao PDR; ^2^ Pediatric Department, Tulane University School of Medicine, New Orleans, LA, USA; ^3^ Pediatric Department, Mahosot Hospital, Vientiane, Lao PDR; ^4^ Nuffield Department of Medicine, Centre for Tropical Medicine, Nuffield Department of Medicine, Churchill Hospital, University of Oxford, UK

**Keywords:** Neonatal sepsis, *S. aureus*, developing countries, Laos, post-partum behavior

## Abstract

As data about the causes of neonatal sepsis in low-income countries are inadequate, we reviewed the etiology and antibiotic susceptibilities of bacteremia in young infants in Laos. As *Staphylococcus aureus* is the leading cause of bacteremia in Lao infants, we also examined risk factors for this infection, in particular the local practice of warming mothers during the first weeks postpartum with hot coals under their beds (hot beds). Clinical and laboratory data regarding infants aged 0–60 days evaluated for sepsis within 72 h of admission to Mahosot Hospital in Vientiane, Laos, were reviewed, and 85 of 1438 (5.9%) infants’ blood cultures grew a clinically significant organism. Most common were *S. aureus, Escherichia coli* and *Klebsiella pneumoniae*. Whereas no methicillin-resistant *S. aureus* was found, only 18% of *E. coli* isolates were susceptible to ampicillin. A history of sleeping on a hot bed with mother was associated with *S. aureus* bacteremia (odds ratio 4.8; 95% confidence interval 1.2–19.0).

## Introduction

Efforts to decrease mortality rates in children under the age of 5 years, as targeted in the United Nations Millenium Development Goal 4, have been more successful in reducing the death rate in older children than in neonates. In 2010, deaths in the neonatal period contributed 40% and 52% of mortality rates in children under the age of 5 years worldwide and in Southeast Asia, respectively [1]. As more than one-third of neonatal mortality is atributable to severe infections, many deaths might be avoided by enhanced interventions to reduce the incidence of neonatal sepsis and improve access to appropriate antibiotic treatment [2–4].

In high-income countries, neonatal sepsis is most often caused by *Streptococcus agalactiae* and *Escherichia coli,* in particular during the first 72 h of life (early-onset sepsis or EOS) [5,6]. The empirical use of penicillin and an aminoglycoside in this age-group is common worldwide. The causes of late-onset sepsis (LOS) are more diverse, and empirical antibiotic protocols vary widely [7,8]. Knowledge about the etiology of neonatal sepsis and pathogen antibiotic susceptibility in low-income countries (LICs) is extremely limited owing to the rarity of culture diagnostic facilities [9]. The available data suggest that leading bacterial causes of sepsis in young infants (0–60 days of life) in LICs include *Staphylococcus aureus, Klebsiella pneumoniae* and *E. coli* [10,11], which are most often not susceptible to ampicillin.

**Fig. 1.**
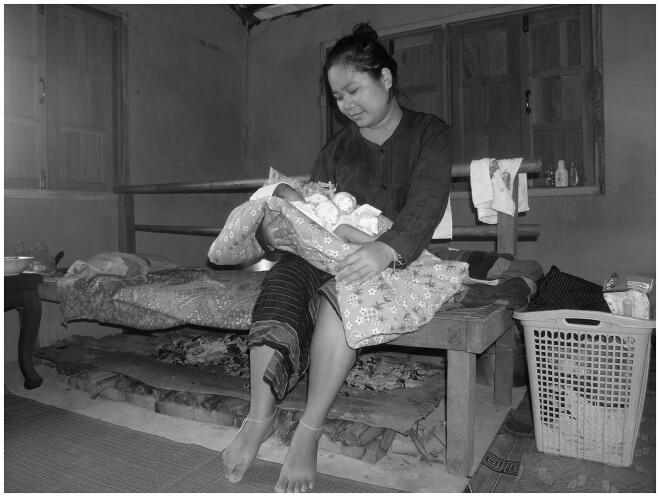
Urban Lao mother caring for her newborn while being warmed by hot coals placed under the bed (the mother gave her written informed consent for publication of this photograph).

The Lao PDR (Laos) has an estimated infant mortality rate of 76/1000 live births [12], but there are limited data on infectious disease epidemiology among Lao infants. In a prospective study of the causes of bacteremia over 4 years in Vientiane, a high proportion (69%) of bacteremia in Lao infants was caused by *S. aureus* [13]. We hypothesized that infants may become more vulnerable to *S. aureus* through the heating of their skin during the Lao postpartum practice of roasting mothers (with their newborns) with hot coals lain underneath their beds (Fig. 1) [14,15]. We therefore reviewed the causes of bacteremia in young infants in Vientiane and attempted to investigate potential risk factors, including infants’ history of hot bed exposure, for *S. aureus* bacteremia.

## Methods

Mahosot Hospital is a 400-bed government hospital in the capital city Vientiane. Each year, the neonatal and pediatric units admit approximately 400 ill or at-risk newborns directly from the obstetrics ward and approximately 100 young infants coming from home or other hospitals. Supplemental oxygen, temperature-controlled incubators, mechanical ventilation and peripheral intravenous catheters are available; umbilical or other central catheters are not. Most costs of hospitalization are borne by patients and families [16].

All admitted infants aged 0–60 days who had a blood culture performed within the first 72 h of their hospitalization between February 2000 and September 2011 were included. Blood cultures had been offered at no cost to the family as part of a larger study of the causes of fever in Laos. A physician’s request for a blood culture on suspicion of sepsis (for which there were no formal guidelines or restrictions) and parental informed consent were the only criteria for enrollment. Ethical approval was granted by the Ethical Review Committee of the Faculty of Medical Sciences, National University of Laos, and the Oxford Tropical Ethics Review Committee.

Clinical information for each patient was collected at the time of sampling and, subsequently, available hospital paper charts were also reviewed. Data obtained by paper chart review were collected on a standardized form by Mahosot Hospital pediatricians.

Blood cultures were performed using standard procedures [13]. The clinical significance of positive cultures was determined by a team of physicians at the time of the result based on factors that included the identity of the organism and the number of samples growing the same organism [17].

Antibiotic susceptibilities were determined by the agar disk diffusion method according to 2010 Clinical and Laboratory Standards Institute guidelines. Extended-spectrum beta-lactamase (ESBL) detection was performed by cefpodoxime screening and confirmation by Clinical and Laboratory Standards Institute-recommended disk diffusion methods [18].

We calculated unadjusted odds ratios with 95% confidence intervals for clinical factors present at the time of blood culture and their association with culture result (clinically significant organism vs. no growth); growth of *S. aureus* vs. any other result and poor outcome (defined as death in hospital or discharged moribund) vs. good outcome (discharged well or discharged ill but improving). Differences in hospital outcome by group, as defined by blood culture results, were assessed with χ^2^ tests. Analysis was performed using STATA 10.1 (Stata Corp, College Station, TX).

## Results

Between 2000 and 2011, 1438 infants from birth to 60 days of age had blood cultures performed within 72 h of hospital admission. Their median (range) age was 3 (0–60) days. Blood cultures from 85 infants (5.9%) grew a clinically significant organism; 19 of 644 (3.0%) infants evaluated for EOS (age listed as 0, 1 or 2 days old), 56 of 630 (8.9%) infants evaluated for LOS (age listed as 3–28 days old) and 10 of 164 (6.1%) infants evaluated in the second month of life (Table 1). The most common organisms were *S. aureus, E. coli* and *K. pneumoniae.* In addition, blood cultures from 198 (13.8%) infants grew a contaminant and 25 (1.7%) grew organisms of unclear significance (Table 1 footnote).

**Table 1:** Causes of culture-confirmed bacteremia among Lao infants admitted to Mahosot hospital

	Early onset (0–2 days of life)	Late onset (3–28 days of life)	Second month (29–60 days of life)	All young infants (0–60 days of life)
	Number (%)	Number (%)	Number (%)	Total (%)
*Staphylococcus aureus*	6 (32)	31 (55)	2 (20)	39 (46)
*Escherichia coli*	3 (16)	5 (9)	3 (30)	11 (13)
*Klebsiella pneumoniae*	4 (21)	5 (9)	0	9 (11)
*Enterobacter aerogenes*	1 (5)	3 (5)	1 (10)	5 (6)
*Enterococcus faecalis*	1 (5)	2 (4)	1 (10)	4 (5)
*Streptococcus pyogenes*	0	2 (4)	1 (10)	3 (4)
*Streptococcus agalactiae*	2 (11)	0	1 (10)	3 (4)
*Streptococcus pneumoniae*	0	2 (4)	1 (10)	3 (4)
*Burkholderia pseudomallei*	0	2 (4)	0	2 (2)
*Acinetobacter baumannii*	0	2 (4)	0	2 (2)
*Listeria monocytogenes*	1 (5)	0	0	1 (1)
*Proteus mirabilis*	1 (5)	0	0	1 (1)
*Pseudomonas aeruginosa*	0	1 (2)	0	1 (1)
*Salmonella* sp.	0	1 (2)	0	1 (1)
Total	19	56	10	85

In addition, blood cultures from 198 (13.8%) young infants grew bacteria classified as contaminants, including 162 CONS, 10 *Bacillus* spp., 6 *Enterobacter* spp., 5 *Pseudomonas* spp., 3 *Klebsiella* spp*.*, 2 *Leuconostoc* spp. and various other environmental gram-negative organisms and likely skin-contaminants. Cultures from an additional 25 infants grew organisms of unclear significance (1.7%). These included 8 *Acinetobacter* spp., 5 Staphylococci of uncertain identity, 4 *Enterococcus* spp., 2 *Streptococcus* spp., 1 *Candida* sp. and 5 other environmental gram-negative organisms

Antimicrobial susceptibilities for the three most common organisms and by patient age-group are shown in Table 2. There were no cases of methicillin-resistant *S. aureus* (MRSA)*.* ESBL-producing *K. pneumoniae* was grown from samples from two infants. Only 18% of *E. coli* isolates were susceptible to ampicillin, but 73% were susceptible to gentamicin.

**Table 2:** Antibiotic susceptibility of clinically significant organisms isolated: % susceptible (# with information)

	*S. aureus*	*E. coli*	*K. pneumoniae*	Infants 0–2 days of life	Infants 3–28 days of life	Infants 29–60 days of life
Ampicillin	5% (39)	18% (11)	0% (9)	35% (17)	13% (55)	33% (9)
Amoxicillin-clavulanic acid	100% (39)	63% (11)	67% (9)	83% (18)	93% (46)	63% (8)
Ceftriaxone	100% (39)	91% (11)	67% (9)	76% (17)	84% (51)	89% (9)
Chloramphenicol	72% (39)	60% (10)	44% (9)	59% (17)	71% (51)	67% (9)
Co-trimoxazole	97% (39)	18% (11)	56% (9)	73% (15)	79% (48)	43% (7)
Erythromycin	59% (39)	–	–	100% (8)	56% (36)	80% (5)
Gentamicin	100% (39)	73% (11)	78% (9)	93% (15)	88% (48)	100% (7)
Ofloxacin	–	100% (11)	100% (8)	91% (11)	100% (13)	100% (7)
Oxacillin	100% (39)	–	–	42% (19)	60% (55)	50% (10)
Penicillin	5% (39)	–	–	33% (9)	14% (36)	60% (5)
Vancomycin	100% (39)	–	–	38% (16)	62% (52)	56% (9)

Clinical and laboratory features associated with growth of a clinically significant organism were history of fever, axillary temperature >38°C, skin infection, jaundice and an absolute neutrophil count (ANC) <1500/mm^3^ (Table 3). Features associated with *S. aureus* bacteremia were the presence of skin infection, jaundice and accompanying mother on hot bed (Table 3).

**Table 3:** Factors associated with culture-proven bacteremia, *S. aureus* bacteremia and poor outcome (unadjusted odds ratio)

Factor (Number of infants with data available)	Culture-proven bacteremia OR (95% CI) (*p* value)	*S. aureus* bacteremia OR (95% CI) (*p* value)	Death or discharged moribund OR (95% CI) (*p* value)
Weight <2.5 kg (1019)	1.4 (0.77–2.4) (0.295)	1.2 (0.48–2.9) (0.713)	**2.4 (1.2–4.8) (0.013)**
History of fever (862)	**3.0 (1.6–5.5) (0.001)**	1.8 (0.80–4.2) (0.156)	0.84 (0.43–1.7) (0.631)
History of antibiotics before blood culture (641)	1.0 (0.43–2.3) (0.966)	0.56 (0.13–2.5) (0.446)	1.2 (0.39–3.5) (0.789)
Hyperthermia ≥38°C axillary (1293)	**2.0 (1.3–3.1) (0.002)**	1.4 (0.73–2.6) (0.316)	1.0 (0.57–1.7) (0.917)
Dyspnea (841)	1.7 (0.9–3.0) (0.068)	1.2 (0.49–2.8) (0.732)	**3.2 (1.5–6.4) (0.002)**
Pallor (1131)	1.2 (0.74–2.2) (0.370)	1.2 (0.55–2.7) (0.631)	**4.0 (2.2–7.3) (<0.001)**
Jaundice (1138)	**1.7 (1.0–2.6) (0.033)**	**2.1 (1.1–4.1) (0.026)**	1.3 (0.72–2.2) (0.412)
Skin infection (1172)	**4.7 (2.9–7.6) (<0.001)**	**7.8 (4.0–15.2) (<0.001)**	1.3 (0.60–2.2) (0.688)
^a^Elevated CRP (131)	3.6 (0.69–19.0) (0.127)	2.3 (0.24–21.7) (0.467)	**20.5 (3.3–125) (0.001)**
^a^Low WBC <5000/mm^3^ (245)	0.71 (0.15–3.3) (0.659)	–	7.2 (0.95–54) (0.056)
^a^Elevated WBC >25 000/mm^3^ (245)	0.92 (0.25–3.3) (0.902)	0.82 (0.34–2.0) (0.660)	2.3 (0.43–12.4) (0.326)
^a^Low ANC <1500/mm^3^ (221)	**4.3 (1.2–15.3) (0.028)**	3.8 (0.92–16.0) (0.065)	**5.0 (1.0–24.4) (0.044)**
^a^Low platelets <100 000/mm^3^ (197)	2.9 (0.78–10.8) (0.114)	1.0 (0.12–8.7) (0.969)	2.3 (0.42–12.1) (0.345)
^a^On hotbed with mother (91)	2.1 (0.64–6.6) (0.226)	**4.8 (1.2–19.0) (0.028)**	1.5 (0.26–8.2) (0.659)

^a^Data primarily collected retrospectively from paper chart review

**Fig. 2.**
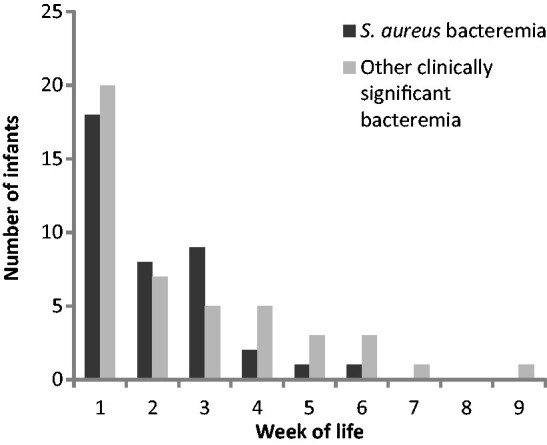
Infant age at diagnosis of *S. aureus* bacteremia vs. all other causes of bacteremia.

There were data about hot bed exposure for 91 infants obtained through chart review. Of note, 57% of infants with *S. aureus* bacteremia had a skin infection noted, in contrast to 31% of those with bacteremia from another organism and 14% of those who had no growth. The frequency of *S. aureus* bacteremia compared with all other clinically significant organisms by week of age is shown in Fig. 2.

Of infants for whom outcome data were available, 79 of 514 (15%) either died in hospital (37) or were discharged moribund (42) at the family’s request. Poor hospital outcome was associated with weight <2.5 kg, dyspnea, pallor, signs of meningitis, an elevated C-reactive protein and an ANC <1500/mm^3^; but not with skin infection or hot bed exposure (Table 3). In comparison with infants with no blood culture growth, poor outcome was associated with growth of a clinically significant organism (Table 4).

**Table 4:** Hospital outcome by group as defined by blood culture results

Group (number with outcome data available)	Death or discharged moribund	*p* value of difference (by chi-squared) from no-growth group
No growth (383)	12%	–
**Clinically significant bacteremia (58)**	**31%**	**<0.001**
Bacteremia with organism of unclear significance (19)	21%	0.262
Growth of presumed contaminant (54)	19%	0.202

## Discussion

Neonates had a high rate of *S. aureus* bacteremia especially in the first 3 weeks of life, even compared with that seen in most other LICs [11]. We found an expected relationship between infant skin infection and *S. aureus* bacteremia.

Having been on the maternal hot bed was also associated with *S. aureus* bacteremia. Whether this is a causal relationship is uncertain. As these data were extracted from hospital records retrospectively, there is a possibility of observer bias confounding the relationship. However, the young age of the infants in whom *S. aureus* was diagnosed also suggests that hot beds may be important in the genesis of *S. aureus* bacteremia by causing skin heat damage (Fig. 2). The normal duration of hot bed use postpartum is 2 weeks [14], and *S. aureus* bacteremia was dominant only until 3 weeks of age*.*

This potential association may be due to the presence of frank burns which become infected, but may also be due to increased ambient temperatures which induce vasodilation and may increase the risk of bacteremia through superficial skin trauma if colonized with *S. aureus* [19]. Although studies of *S. aureus* colonization show that few (3–5%) infants are colonized with *S. aureus* at birth, horizontal acquisition has been shown to occur quickly, with reported colonization rates as high as 19% by day 3 of life [20,21].

Maternal hot bed use is widespread in East and Southeast Asia, in Laos, Thailand, Burma, the Philippines, China, Vietnam, Cambodia and Malaysia [22–24]. The potential negative consequences have been little discussed. Prospective studies of hot bed behavior and exposure, the temperatures infants may be exposed to, infant skin findings and bacteremia may help to clarify this possible relationship. If this association is substantiated by further prospective studies, adaptations to maternal hot bed behavior that may be acceptable to mothers and their families but reduce the risk of neonatal *S. aureus* sepsis could be considered. Investigations of the incidence of *S. aureus* sepsis in other communities in Southeast Asia where hot bed use is common are needed. Whereas MRSA remains extremely rare in Vientiane, community-acquired MRSA is an important clinical problem in adjacent Cambodia [25]. It would be beneficial to identify any potential effective interventions to reduce *S. aureus* bacteremia in infants before community-acquired MRSA becomes a problem in Laos.

Limitations of the study include a population that was selected from an urban referral hospital and thus different from that of most of Laos where access to hospital care for sick infants is variable [15]. Missing data and use of retrospective chart review are additional serious limitations. We have not determined whether infants were infected with Panton-Valentine leukocidin-positive *S. aureus*, often associated with skin infections [25]. The choices made by the team in labeling organisms ‘clinically significant’, ‘contaminant’ or ‘of unclear significance’ were naturally subject to error, but we chose to retain the categorization given by the team at the time of each infant’s evaluation rather than second-guess those decisions years later. Our outcome data (Table 4) seem to support the accuracy of those decisions.

Despite these limitations, these data are helpful in informing local antibiotic choice. The high rate of *E. coli* ampicillin resistance noted in our population is concerning, as is the presence of ESBL producers [26]. Attempts to alter or escalate empiric therapy guidelines in response to each change in the local pattern of bacterial pathogens and their susceptibilities have met with mixed results [27]. In response to increasing rates of ampicillin resistance in the 1990s, some neonatal units in high-income countries changed empirical neonatal sepsis treatment policy to include a cephalosporin (with or without ampicillin or an aminoglycoside) [28,29]. Whereas some hospitals reported improvement in mortality rates with this change [29,30], others noted an increase in candidemia and ESBL-producing Enterobacteriaceae infections [31,32].

Changing national empirical treatment guidelines for neonatal sepsis to expensive and broad-spectrum antibiotic combinations is not a feasible solution for low-income settings. It would also risk worsening current challenges with antimicrobial resistance. For Vientiane neonates, the most prudent and cost-effective course might be continuing use of ampicillin (or penicillin) and gentamicin for EOS, except when a staphylococcal infection is suspected, when substituting cloxacillin for ampicillin would be preferred. For LOS, we suggest cloxacillin and gentamicin with the addition of a third-generation cephalosporin in those with suspected meningitis [8].

For second-line therapy (in the case of neonates who continue to show signs of sepsis after 48 h of empiric therapy, but who do not have access to blood culture results to guide therapy), our results suggest that either amoxicillin/clavulanic acid, a quinolone, or a third-generation cephalosporin such as ceftriaxone or cefotaxime (all of which penetrate the CSF well) would be a reasonable next step. Infants older than 1 month with signs of sepsis and/or meningitis could be reasonably treated with ceftriaxone, which is sufficiently broad-spectrum, relatively inexpensive and convenient to administer. Without current evidence for MRSA bacteremia, the use of vancomycin is not indicated. Increasing antibiotic resistance could prove to be a devastating health and economic problem in Laos, and antibiotic stewardship and hospital hygiene need to be high priorities.

No laboratory test was sufficiently sensitive to predict bacteriologically confirmed sepsis in this population. Having a low ANC was associated with confirmed bacteremia as well as poor outcome, but a relatively small proportion of infants (11/257) had a low ANC.

Randomized controlled trials of alternative empirical antibiotic strategies are urgently needed to guide the management of young infants with signs of a serious bacterial infection in LICs. Trials of community rectal, oral or intra-muscularly administered antibiotics by local health workers in rural Laos may provide vital information for reducing infant mortality [4]. It is likely that many ill infants from remote areas die without the evaluation or care of any health worker [33].

Increasing access to blood culture testing for all sick Lao infants will not likely be feasible or cost-effective in the near future. However, continued surveillance of selected infants with clinical sepsis in a variety of settings, including rural areas, is necessary for informing national treatment guidelines as well as keeping physicians aware of local antibiotic resistance trends.

## Funding

This work was supported by the Wellcome Trust of Great Britain.
